# Elevated Hedgehog activity contributes to attenuated DNA damage responses in aged hematopoietic cells

**DOI:** 10.1038/s41375-019-0641-3

**Published:** 2019-11-14

**Authors:** Annika Scheffold, Ali H. Baig, Zhiyang Chen, Sarah E. von Löhneysen, Friedrich Becker, Yohei Morita, Alush I. Avila, Marco Groth, André Lechel, Florian Schmid, Johann M. Kraus, Hans A. Kestler, Stephan Stilgenbauer, Melanie Philipp, Martin D. Burkhalter

**Affiliations:** 1 Department of Internal Medicine III, University Hospital Ulm, 89081 Ulm, Germany; 2 Leibniz Institute on Aging, Fritz Lipmann Institute, 07745 Jena, Germany; 3 Department of Internal Medicine I, University Hospital Ulm, 89081 Ulm, Germany; 4 Institute of Medical Systems Biology, Ulm University, 89081 Ulm, Germany; 5 Institute of Biochemistry and Molecular Biology, Ulm University, 89081 Ulm, Germany; 6 Department of Experimental and Clinical Pharmacology and Pharmacogenomics, Division of Pharmacogenomics, University of Tübingen, 72074 Tübingen, Germany

**Keywords:** Haematopoietic stem cells, Cell signalling

## Abstract

Accumulation of DNA damage and myeloid-skewed differentiation characterize aging of the hematopoietic system, yet underlying mechanisms remain incompletely understood. Here, we show that aging hematopoietic progenitor cells particularly of the myeloid branch exhibit enhanced resistance to bulky DNA lesions—a relevant type of DNA damage induced by toxins such as cancer drugs or endogenous aldehydes. We identified aging-associated activation of the Hedgehog (Hh) pathway to be connected to this phenotype. Inhibition of Hh signaling reverts DNA damage tolerance and DNA damage-resistant proliferation in aged hematopoietic progenitors. Vice versa, elevating Hh activity in young hematopoietic progenitors is sufficient to impair DNA damage responses. Altogether, these findings provide experimental evidence for aging-associated increases in Hh activity driving DNA damage tolerance in myeloid progenitors and myeloid-skewed differentiation. Modulation of Hh activity could thus be explored as a therapeutic strategy to prevent DNA damage tolerance, myeloid skewing, and disease development in the aging hematopoietic system.

## Introduction

Given the pivotal role of stem cells in tissue maintenance and cancer formation, the protection of stem cells from DNA damage appears to be of utmost importance for the evolution of long-lived vertebrate species. One measure of stem cell protection from environmental factors inducing DNA damage certainly is the sheltering in niches [1]. Keeping stem cells in a quiescent state with a slow cell cycle profile and markedly reduced metabolic activity further evolved as a protective mechanism against accumulation of DNA damage (reviewed in ref. [2]). The hematopoietic system is particularly sensitive to defects in molecular pathways governing DNA repair and genomic stability, which is reflected in premature exhaustion of hematopoietic stem cells (HSCs) upon failure of genome integrity maintenance systems [3–6]. This is also illustrated by an accumulation of markers for DNA damage in HSCs of normally aging, repair-proficient mice and men [5, 7], although the nature of the origin for this accumulation is under debate and may involve defects in resolving the persistence of phosphorylated DNA damage response factors [8].

Aging-associated accumulation of DNA breaks in hematopoietic stem cells (HSCs) as well as progenitor cells may contribute to the emergence of mutant, clonal hematopoiesis of indeterminate potential (CHIP) [9]. CHIP can be detected in peripheral blood of up to 50% of elderly humans associating with evolution of hematologic disorders and increases in mortality [10–13]. In addition, DNA damage aggravates skewed differentiation in the aging hematopoietic system towards a decrease in lymphopoiesis and an increase in myelopoiesis associating with drifts in the HSC pool harboring an increasing percentage of myeloid-biased vs. lymphoid-biased HSCs [14–17].

Mechanistically, the causes for increases in DNA damage in aging HCSs and progenitors remain incompletely understood, but could be explained by defects in checkpoint responses. Recently, it has been shown that activation of ATM and apoptosis-priming is reduced in aged HSCs in the context of different types of DNA damage, especially DNA double-strand breaks (DSBs) [18]. These deficiencies affect both myeloid- and lymphoid-biased HSCs, yet do not lead to impairments in DNA repair kinetics of DSBs [18]. Checkpoints that specifically respond to DNA damage in lymphoid-biased HSCs have been identified and include activation of the regulator of circadian gene expression, Per2, and the differentiation inducing transcription factor, Batf [19, 20]. Whether there are aging-associated alterations that affect DNA damage responses (DDRs) specifically in myeloid-biased HSCs and myeloid progenitors is currently unknown. Such alterations could influence the evolution of myeloid-skewed differentiation and mutation selection in aging HSCs and progenitors leading to CHIP.

Here, we show that aging renders murine hematopoietic progenitors more resistant to DNA damage, in particular bulky DNA adducts. Transcriptome analysis revealed upregulation of *Evc* and *Evc2* specifically in myeloid progenitors and myeloid-biased HSCs, what coincided with elevated Hedgehog (Hh) signaling activity in such cells. Manipulation of Hh signaling activity using pharmacological as well as short-hairpin RNA (shRNA)-based approaches showed that Hh signaling by itself is sufficient to drive DNA damage-resistant proliferation of progenitors from young mice, whereas Hh inhibition restores DDRs and abrogates elevated rates of DNA damage-resistant proliferation of hematopoietic progenitors from old mice. Altogether, these data suggest that age-related elevation of Hh activity and DNA damage-resistant proliferation of myeloid-progenitor cells contributes to the development of myeloid-skewed hematopoiesis in aged mice.

## Materials and methods

The goal of this study was to analyze the intrinsic capability of aged hematopoietic cells to cope with bulky DNA lesions. We therefore freshly extracted and isolated different types of hematopoietic cells from C57Bl/6J wild-type mice of different age and both sexes. Young mice had an age of 12–14 weeks, while aged mice were at least 22 months old. The freshly isolated cells were then subjected to different treatments to induce bulky DNA lesions. Different experimental approaches were then applied such as colony formation assays, Western blotting, or analysis of DNA repair activities to obtain insight how treated cells react to bulky DNA lesions. Please find detailed information about experimental procedures in the supplement.

## Results

### Aging hematopoietic cells show higher tolerance for bulky DNA adducts

Defects in nucleotide excision repair (NER) have been shown to induce HSC failure and segmental progeria of the hematopoietic system [4, 5]. Moreover, maintenance of HSCs highly depends on the expression of Aldh2, which prevents endogenous aldehydes to induce DNA lesions. These are typically repaired by NER and the combined inactivation of NER and aldehyde defense leads to premature bone marrow failure [21]. Despite the importance of NER for the maintenance of the hematopoietic system, knowledge about changes in the functionality of this system in the context of natural aging is still lacking. We therefore decided to challenge NER in hematopoietic progenitor cells (lineage marker-negative bone marrow cells; Lin- cells) from young and old mice by induction of bulky adducts, which represent the specific substrate for NER (reviewed in ref. [22]). Such bulky DNA lesions can be formed by diverse mechanisms and agents. Ultraviolet (UV) irradiation is the best characterized inducer of bulky lesions in the lab or exposed tissues, however, cisplatin and its derivatives, polycyclic aromatic hydrocarbons, or endogenous aldehydes can be sources of this class of damage, too, which even reach sheltered stem cells (reviewed in ref. [22]). Importantly, NER recognizes damage based on distortion of the DNA helix and stalling of RNA polymerases, regardless of the actual chemical property of the lesion [23–26]. We chose UVC (254 nm) irradiation for induction of lesions due to its ease of handling and reproducibility.

**Fig. 1:**
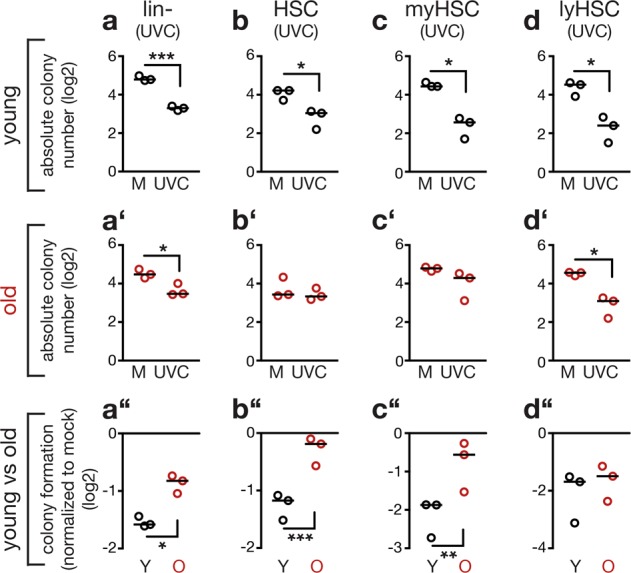
Aging alleviates suppression of colony formation of hematopoietic stem and progenitor cells in response to bulky DNA adduct induction. The scatter plots depict the capacity of the indicated types of hematopoietic stem and progenitor cells from young and old mice to form colonies in response to UVC. All cells were freshly isolated, left untreated or irradiated with 7.5 J/m^2^ UVC and growth of colonies was assessed 10 to 14 days later. Shown are **(a** to **d’)** absolute numbers of formed colonies as well as the **(a”** to **d”**) comparison of colony number between control and treated cells isolated from individual mice. Individual data points represent results obtained with cells isolated from individual mice (biological replicates). Data were log-transformed to obtain normal distribution. Black lines: median. Two-tailed unpaired Welch’s *t-*test (**a** to **d’**) and two-tailed paired *t*-test (**a”** to **d”**). **a**, **a’**, **a”** Lineage-negative (Lin-) cells from three young and three old mice; *p* = 0.0001 (**a**); *p* = 0.0229 (**a’**); *p* = 0.0112 (**a”**). **b**, **b’**, **b”** Long-term (LT) HSCs from three young and three old mice; *p* = 0.0322 (**b**); *p* = 0.0002 (**b”**). **c**, **c’**, **c”** Myeloid (my)-biased HSCs from three young and three old mice; *p* = 0.0185 (**c**); *p* = 0.0077 (**c”**). **d**, **d’**, **d”** Lymphoid (ly)-biased HSCs from three young and three old mice; *p* = 0.0162 (**d**); *p* = 0.0336 (**d’**)

We exposed Lin- cells from young and old mice to a low dose of UVC (7.5 J/m^2^) and compared their ability to form colonies to control-treated cells. Irradiation suppressed colony formation of Lin- cells from both young and old mice compared to mock-treated cells (Fig. 1a, a’). However, the level of suppression of the colony forming capacity was reduced for Lin- from old mice compared to young mice (Fig. 1a”). Repeating this analysis with highly purified long-term (LT) HSCs (CD34^−^, Lin-, Sca-1^+^, c-Kit^+^ (CD34^-^ LSK); for gating strategy see Supplemental Fig. 1) from young and old mice confirmed this result. When exposed to UV-induced DNA damage vs. mock treatment, HSCs from old mice showed a reduced suppression of the colony forming capacity compared to HSCs from young mice (Fig. 1b, b”).

During aging, hematopoiesis is skewed towards the myeloid lineage due to increases in myelopoiesis resulting in relative reduction of lymphopoiesis [16, 27]. This phenotype coincides with a dominance of myeloid-biased HSCs, preferentially reconstituting the myeloid lineage as opposed to lymphoid-biased HSCs, which preferentially give rise to the lymphoid lineage. High- and low-expression levels of the surface marker CD150 have previously been used to discriminate between myeloid- and lymphoid-biased HSCs [14, 17]. To test whether aging-induced UV resistance affects the entire HSC pool, we therefore assessed the colony forming capacity of myeloid-biased HSCs (CD150^hi^ CD34^-^ LSK; Fig. 1c, c”) and lymphoid-biased HSCs (CD150^low^ CD34^-^ LSK; Fig. 1d, d”) after UV treatment. Only myeloid-biased HSCs exhibited an aging-associated resistance to UV-induced suppression of colony formation (Fig. 1c”). We observed a similar result, when we used carboplatin, a clinically relevant drug, to induce bulky adducts (Supplemental Fig. 2). These data indicate that myeloid-biased HSCs gain resistance to bulky DNA damage during aging.

### DNA damage checkpoint function is attenuated in aged hematopoietic cells

**Fig. 2:**
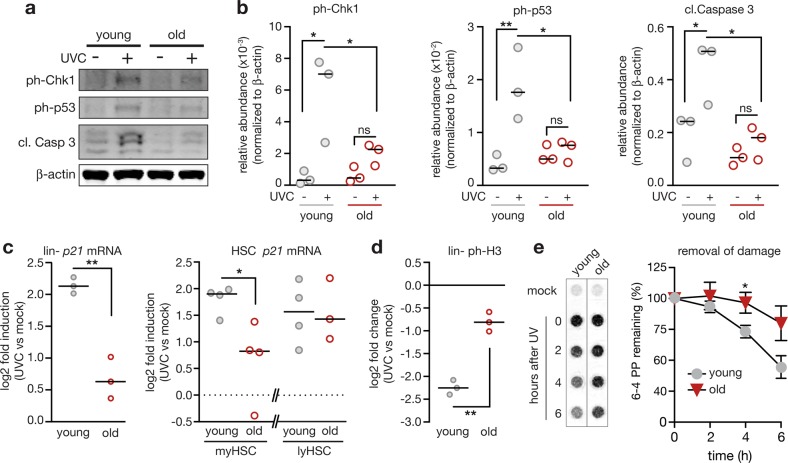
Hematopoietic stem and progenitor cells from aged mice exhibit intrinsic impairments in DNA damage responses (DDRs), delayed DNA repair, and DNA damage-resistant cell cycle activity. **a–c** Checkpoint responses were analyzed in lineage-negative (Lin-) cells and HSCs from young and old mice. Cells were freshly isolated and immediately irradiated with 10 J/m^2^ UVC ( + UVC) or left untreated (-UVC) and lysed 5 h later. **a**, **b** Western blot analysis revealed reduced activation of Chk1 (phosphorylation at serine 345) and p53 (phosphorylation at serine 15) as well as attenuated cleavage of caspase 3. Cells of individual mice (three young and old) were lysed and analyzed separately on the same membrane. **a** Representative western blot. Scans of whole membranes are shown in Supplemental Fig. 3. All antigens were detected on one membrane from the identical transfer. **b** Signals were quantified after normalization to β-actin. Two-way ANOVA with Holm-Sidak correction. Young mock vs. UVC *p* = 0.0111 (ph-Chk1), *p* = 0.0076 (ph-p53), *p* = 0.0236 (cl. Casp. 3); UVC young vs old *p* = 0.0470 (ph-Chk1), *p* = 0.0164 (ph-p53), *p* = 0.0169 (cl. Casp. 3).  Bar indicates the median. **c** The histograms show the qPCR analysis of *p21* mRNA levels normalized to *Hmbs* (Lin- cells) or *Polr2a* (HSCs) in freshly isolated Lin- cells (*n* = 3), myeloid-biased HSCs (*n* = 4) and lymphoid-biased HSCs (*n* = 4 and 3) from aged mice compared to young control mice. Lysates of cells from individual mice were analyzed separately and are depicted as data points (circles). Fold induction levels of *p21* mRNA were calculated by comparing irradiated vs. mock-treated cells from the same mouse. Black bars represent median. Data was log transformed. Two-tailed Welch’s test: Lin- cells: *p* = 0.0087; myHSCs: *p* = 0.0486. **d** Freshly isolated Lin- cells were left untreated or irradiated with 10 J/m^2^ UVC, fixed 5 h later, and probed for levels of phospho-Histone H3 (ph-H3) as measure for mitotic cells. Cells of individual mice were analyzed separately. Three mice per age group were analyzed. Circles reflect data obtained from individual mice. Fold changes of ph-H3 levels were calculated by comparing levels of irradiated with mock-treated cells that were isolated from the same mouse. To obtain normal distribution, data was log transformed. Two-tailed Welch’s test: *p* = 0.001. Bar indicates the median **e** Bulky DNA adducts, specifically 6–4 photoproducts (6–4 PP), were measured at indicated time points after UV-irradiation (20 J/m^2^ UVC) or control treatment. Freshly isolated, Lin- cells from 2-month or 24-month-old mice were employed (*n* = 4 mice per group); two-tailed, Welch’s *t-*test, young vs. old, 4 h *p* = 0.0460, 6 h *p* = 0.0513. The depicted signal intensities are derived from the same membrane (Supplemental Fig. 3). Mean ± SEM

To analyze whether aging changes DDRs to bulky DNA lesions, we determined levels of activated Chk1 and p53 (phosphorylated at serine 345 and serine 15, respectively) in UV-irradiated Lin- cells from young and old mice (Fig. 2a, b and Supplemental Fig. 3). This analysis revealed a reduced activation in old cells of both Chk1 and p53, respectively. Furthermore, induction of apoptosis as estimated by levels of cleaved caspase 3 was attenuated in Lin- cells from old mice (Fig. 2a, b). Similarly, messenger RNA (mRNA) induction of the p53 target gene, *Cdkn1a* (*p21*), was reduced in UV-irradiated Lin- cells from old compared to young mice (Fig. 2c). The analysis of UVC-dependent *p21* mRNA induction in highly purified subpopulations of HSCs revealed that especially myeloid-biased HSCs show an age-related decrease of *p21* induction while lymphoid-biased HSCs seem unaffected (Fig. 2c). Altogether, these data suggest that aging impairs checkpoint responses to bulky DNA lesions in myeloid-biased HSCs and myeloid progenitors. This failure in checkpoint activation correlated with increases in the percentage of Lin- cells entering G2/M stages of the cell cycle in UV-irradiated cells from old mice compared to cells from young mice (Fig. 2d and Supplemental Fig. 4). Measurement of UV-induced DNA lesions at different time points after irradiation revealed longer persistence of DNA damage in Lin- cells from old compared to young mice (Fig. 2e and Supplemental Fig. 3). This indicates  that DNA repair was delayed and the cells from old mice continued genome duplication despite the presence of damaged DNA.

### Elevated expression of the Evc complex in aged common myeloid-progenitor cells

We next wished to identify candidate pathways causing aging-related impairments of UV-induced DDRs in myeloid-progenitor cells. In order to obtain sufficient material for an analysis, we turned to common myeloid-progenitor cells (CMPs; Lin-, cKit^+^, Sca-1^−^, CD34^+^, FcγR^low^) and determined the transcriptome of CMPs extracted from young and old mice (Supplemental Fig. 5a; data deposited in Gene Expression Omnibus (GSE74093)). Strikingly, the genes showing second and sixth highest age-associated upregulation (of 654 at least 1.5-fold significantly upregulated genes) encode proteins that together form a heterodimeric complex, *Ellis van Crefeld gene syndrom* (*Evc*) and *Ellis van Crefeld gene syndrom 2 (Evc2)* (Supplemental Fig. 5a). The complex of the Evc and Evc2 proteins positively regulates activity of the Hedgehog (Hh) signaling pathway and mutations in either factor triggers phenotypes that typically are observed in patients showing aberrant Hh signaling capacity [28, 29]. The complex ensures trafficking of *smoothend* (SMO), the central activating component of the Hh pathway to the correct cellular localization [28, 29]. Quantitative PCR (qPCR) analysis also showed an upregulation of *Evc* and *Evc2* in freshly isolated Lin- cells from old vs young mice (Supplemental Fig. 5b, c). The analysis of CMPs and common lymphoid progenitors (CLPs; Lin-, Sca-1^low^, cKit^low^, Flt3^+^, IL-7R^+^) revealed that the age-related increase in the expression of these genes was predominantly observed in CMPs (Supplemental Fig. 5d–g). Furthermore, immunofluorescence staining of Evc2 protein on freshly isolated myeloid- and lymphoid-biased HSCs revealed an age-related increase of expression in myeloid-biased HSCs, but not in lymphoid-biased HSCs (Supplemental Fig. 5h, i). Altogether, *Evc*/*Evc2* expression is upregulated in the myeloid branch of the aging hematopoietic system.

### Altered epigenetic control causes elevated expression of the Evc complex

Since there is increasing evidence that aging-associated alterations in the epigenome contribute to increases in self-renewal of HSCs during aging [30, 31], it was conceivable that epigenetic modifications may contribute to the activation of *Evc*/*Evc2* expression in aged hematopoietic progenitors. Expression of *Evc* and *Evc2* is controlled by a short conserved region (1.6 kb in mice) containing the promoters of both genes (Supplemental Fig. 6a). Histone modifications in this circumscribed region enhance expression of both genes in various types of human leukemia [32]. Chromatin-immunoprecipitation (ChIP) on Lin- cells revealed an aging-associated reduction in repressive marks (H3K27-trimethylation), while activating H3K4-trimethylation marks were unchanged (Supplemental Fig. 6b). Bisulfite sequencing analysis on LSK cells (Lin-, Sca-1^+^, c-Kit^+^) revealed a simultaneous decrease of methylated CpGs in both promoters (Supplemental Fig. 6c, d). Altogether, these data suggest that the loss of epigenetic silencing marks contributes to the aging-associated induction of the expression of *Evc* and *Evc2* in hematopoietic progenitors.

### Age-related induction of Hh signaling activity mainly occurs is myeloid-progenitor cells

**Fig. 3:**
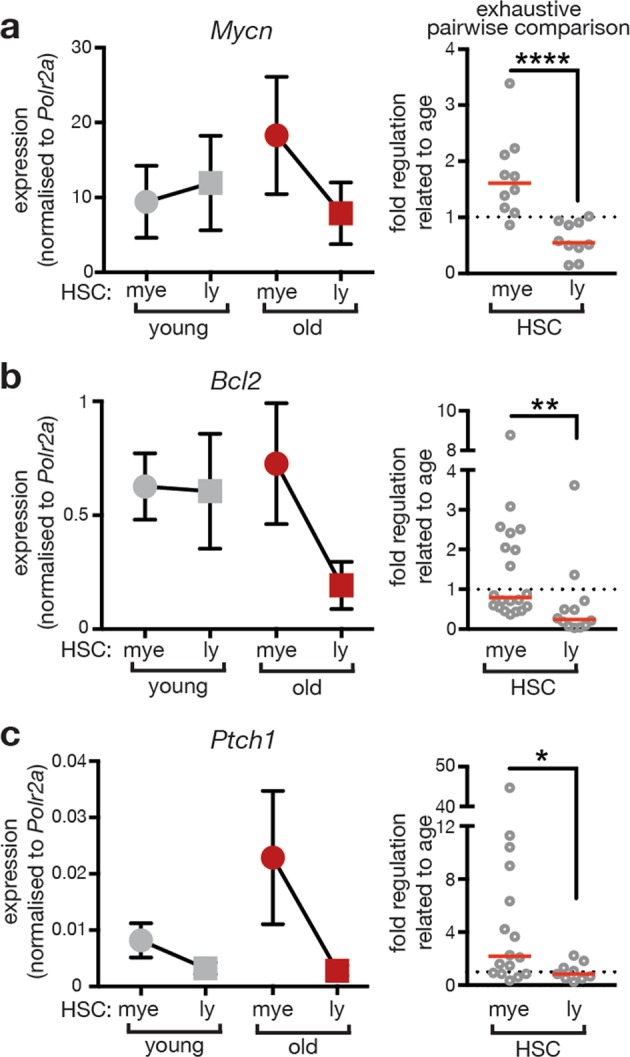
Aging associates with increased expression of Hh-target genes predominantly in myeloid-biased HSCs. Expression levels of Hedgehog target genes *Mycn* (**a**), *Bcl2* (**b**), and *Ptch1* (**c**) were analyzed in myeloid- (mye) and lymphoid- (ly) biased HSCs and normalized to *Polr2a* expression. Left side: Expression levels observed in cells from young and old mice are represented in gray and red, respectively. Lines connect levels determined in young and old mice, respectively. Right side: Exhaustive pairwise comparison was applied to assess aging-related expression changes in myeloid- and lymphoid-biased HSCs. All possible ratios between levels observed in individual old and young mice were calculated and indicated by circles. Mann–Whitney test was used for statistical analysis. Numbers indicate number of mice per age group (young/old): **a** young *n* = 4/4, old *n* = 5/5, *p* < 0.0001 **b** young *n* = 4/3, old *n* = 5/4, *p* = 0.0036 **c** young *n* = 4/3, old *n* = 4/3, *p* = 0.0196. Cells of individual mice were analyzed in technical duplicates or triplicates. Mean ± SEM of biological replicates is represented in graphs on the left. Red line indicates median in graphs on the right. Note that the expression of Hh-target genes increases (*Mycn* and *Ptch1*) or stays stable (*Bcl2*) in myeloid-biased HSCs but declines in lymphoid-biased HSCs during aging

The Evc complex confers Hh signaling [28, 29], suggesting that Hh signaling activity could be increased in aged myeloid-biased HSCs and myeloid progenitors. Indeed, bioinformatics re-sampling analysis of the CMP transcriptome data (Supplemental Fig. 5a) revealed enrichment in Hh pathway components and target genes among the genes showing aging-induced changes (Supplemental Fig. 7a). Additionally, an analysis of published data sets on the transcriptome of HSCs from young vs. old mice [8, 27, 33–35] revealed evidence for a higher number of upregulated than downregulated genes in the Hh pathway in 4 of the 5 data sets (Supplemental Figs. 7b and 8). Moreover, the analysis of mRNA expression levels of exemplary Hh-target genes such as *Ptch1* [36], *Mycn* [37], and *Bcl2* [38] by qPCR analysis suggests upregulated Hh activity in aged Lin- cells and CMPs, whereas CLPs showed a tendency to the opposite (Supplemental Fig. 9). To test whether aging-associated Hh activation occurred in subsets of the HSC pool, we analyzed expression of the same Hh-target genes in myeloid- and lymphoid-biased HSCs (Fig. 3). In cells from young mice target genes were similarly expressed in myeloid- and lymphoid-biased HSCs (gray symbols in Fig. 3). However, Hh-target gene expression was imbalanced in cells from aged mice showing increases in myeloid-biased HSCs compared to lymphoid-biased HSCs (red symbols in Fig. 3). We then compared age-related changes of expression via exhaustive pairwise combination specifically in myeloid- or lymphoid-biased HSCs, respectively [39, 40]. This analysis suggests elevated (*Ptch1* and *Mycn*) or maintained (*Bcl2*) Hh-target gene expression in myeloid-biased HSCs, while expression of these genes was rather reduced in aging lymphoid-biased HSCs (Fig. 3). Altogether, these data suggest that an induction of Hh signaling activity predominantly affects myeloid-biased HSCs and progenitor cells of the myeloid branch of aging mice.

### Manipulation of Hh signaling activity is sufficient to alter the response to bulky DNA lesions

It has previously been shown that Hh pathway activity has the potential to overrule DNA damage checkpoints in cancerous and non-transformed cells [41–45]. We therefore asked whether altered Hh signaling activity contributes to a dysfunction of UV-induced DDRs in hematopoietic progenitor cells. We applied an shRNA-based approach on LSK cells from young and old mice to manipulate Hh pathway activity. Freshly isolated LSK cells from young mice were transduced with lentiviral particles conferring shRNAs targeting *Ptch1*— the central negative regulator of Hh signaling (reviewed in ref. [36]) (Supplemental Fig. 10). Two different shRNAs induced a knockdown of *Ptch1* expression resulting in enhanced Hh activity compared to controls. Of note, instructed enhancement of Hh activity in LSK cells from young mice blunted the activation of p53-dependent DNA damage checkpoints. This furthermore promoted DNA damage-resistant colony formation after induction of bulky DNA lesions via UV-irradiation and aldehyde treatment (Supplemental Fig. 10).

To test whether reduction of Hh signaling activity would revert aging-associated resistance to bulky adducts of LSK cells from old mice, freshly isolated LSK cells from aged mice were targeted with two different shRNAs against *Smo*, the main signal transducing molecule of the Hh pathway (reviewed in ref. [36]). Both shRNAs reduced the expression of Smo, leading to attenuated Hh signaling activity in LSK cells compared to cells transduced with a control shRNA (Supplemental Fig. 10). Instructed reduction of Hh signaling activity was sufficient to partly restore the UV-dependent induction of p53 checkpoint responses and to reinforce the suppression of colony formation of LSK cells from old mice exposed to UV or aldehydes (Supplemental Fig. 10).

**Fig. 4:**
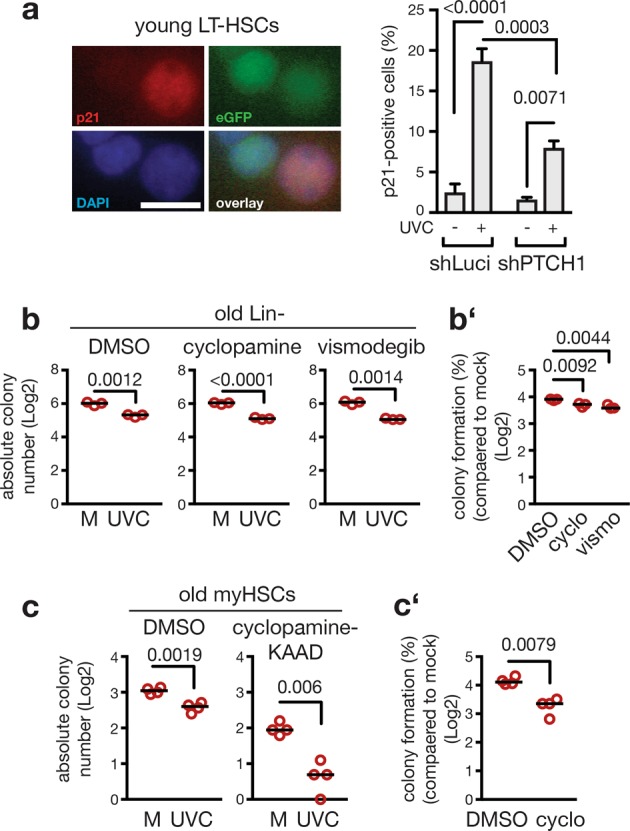
Treatment with Hh inhibitors restores colony formation of aged hematopoietic cells exposed to bulky DNA damage induction. **a** Hh signaling activity in freshly isolated LT-HSCs from young mice was induced via lentiviral transfection of an shRNA against *Ptch1* (shPTCH1) or a control shRNA (shLuci). p21 induction was measured by immunofluorescence 5 h after UV-irradiation (10 J/m^2^) or in non-irradiated controls. eGPF expression marked transfected cells. **a** Representative photographs; the scale bar represents 1 µm. The percentage of p21-positivity in transfected cells was calculated by comparison to the Dapi signal. Two-way ANOVA with Holm-Sidak correction. *n* = 3 mice per group, *n* = 636/623/726/799 nuclei per group; Shown is the mean and SEM; *p* values are indicated. **b**, **b'** Freshly isolated lin- cells from three old mice were treated during 24 h with vehicle (DMSO), cyclopamine (2.5 µM), or vismodegib (5 µM) to inhibit Hh signaling activity. After UVC irradiation (7.5 J/m^2^), cells were plated in methylcellulose in presence of vehicle or inhibitors and colony formation observed 7 days later. Shown are absolute numbers of formed colonies (**b**) as well as the comparison of colony number between control (M) and treated (UVC) cells harvested from individual mice (**b'**). Individual data points represent results obtained with cells harvested from individual mice and thus reflect biological replicates; *n* = 3 mice per group. Two-tailed Welch’s *t*-test (**b**) and one-way ANOVA with Holm-Sidak correction (**b'**) were applied. **c, c'** Freshly isolated myeloid-biased HSCs from four old mice were treated during 24 h with vehicle (DMSO) or cyclopamine-KAAD (2.5 µM), a potent derivative of cyclopamine. After UVC irradiation (7.5 J/m^2^), cyclopamine-KAAD was washed out and colony formation observed 10 days later. Shown are absolute numbers of formed colonies (**c**) as well as the comparison of colony number between control and treated cells harvested from individual mice (**c'**). Individual data points represent results obtained with cells harvested from individual mice and thus reflect biological replicates; *n* = 4 mice per group. **b** to **c’** Data were log transformed. Black lines indicate median; *p* values are indicated in figure

Hence, we hypothesized that manipulation of Hh signaling potentially alters DDRs in HSCs in a similar fashion. Therefore, we promoted Hh pathway activity by knock down of Ptch1 in freshly isolated HSCs (CD34- LSK) from young adult mice followed by induction of bulky DNA adducts via UVC irradiation. Interestingly, HSCs carrying the shRNA against Ptch1 showed a reduced induction of p21 in response to UV-irradiation compared to non-infected cells from the same culture dish (Fig. 4a).

### Small-molecule Hh inhibitors rescue sensitivity of aged hematopoietic cells to bulky DNA adducts

From our results so far we concluded that Lin- cells from aged mice are deficient in DNA damage checkpoint induction, which entails uncontrolled colony formation in response to bulky DNA lesion induction (Figs. 1 and 2). To test whether this effect is due to aging-associated elevation of Hh activity, we pharmacologically inhibited Hh signaling activity in Lin- cells from old mice through administration of cyclopamine or vismodegib, or applied only the vehicle (DMSO). We then analyzed the colony formation capacity after induction of bulky lesions of such treated cells (Fig. 4b). UVC irradiation always caused a reduced number of colonies. However, normalization to the respective mock-treated cells (M) revealed that aberrant colony formation in the presence of DNA lesions was significantly reduced when the Hh pathway was blocked (Fig. 4b’). To test whether this is also the case for myeloid-biased HCSs from old mice, we pretreated such cells with cyclopamine-KAAD, a potent derivate of cyclopamine and assessed the colony formation capacity after induction of bulky lesions. Treatment with the potent cyclopamine-KAAD by itself reduced colony formation compared to control (DMSO) treated cultures (Fig. 4c). This suggests that upregulation of the pro-proliferative Hh pathway helps aged myeloid-biased HSCs to form colonies in culture. However, correction for this effect by normalization to the non-irradiated control (M) revealed that pharmacological inhibition of Hh activity reduced the ability of aged myeloid-biased HSCs to form colonies upon UV-irradiation (Fig. 4c’). Taken together, these data indicate that attenuation of pathologically elevated Hh activity in old myeloid-biased HSCs and myeloid progenitors at least partially restores adequate responses to bulky DNA lesions.

## Discussion

Here, we report that aging triggers elevated Hh signaling activity in the hematopoietic system, particularly in myeloid-biased HSCs and myeloid progenitors. Functionally, this suppresses adequate DDRs after induction of bulky DNA adducts and promotes DNA-damage-resistant proliferation, leading to increased colony formation of cells of the myeloid branch.

Hh signaling is well known for its roles in embryonic development and carcinogenesis (reviewed in ref. [36]). Basal cell carcinoma is a classic example of a cancer type that is caused by elevated Hh signaling activity [46] and Hh inhibition is an accepted strategy to treat this type of cancer [47]. Although Hh signaling was reported to be dispensable for normal hematopoiesis in the young organism [48, 49], overactivation of Hh signaling accelerates regeneration of the hematopoietic system in an experimental transplantation setting [50]. Hematologic disorders are further connected to aberrant Hh signaling activity. In this case, maintenance of leukemic stem cells depends on the Hh pathway [51–55]. Here, we find Hh signaling activity mainly to be elevated in aged myeloid-biased HSCs and myeloid progenitors. We hence conclude that predominantly this branch benefits from the pro-proliferative action of Hh signaling. In the old individual, this could possibly result in a self-propelled expansion of the myeloid branch and promote myeloid skewing. Whether such bias towards the myeloid branch accounts for the decreased HSCs functionality or is actually triggered by it remains to be elucidated. It is certainly possible that the aging hematopoietic system attempts to counteract declined stem cell function by increased production of HSCs in order to match the need of mature hematopoietic cells. Yet, upregulated Hh signaling activity brings the aging hematopoietic system a considerable step closer to a leukemic hematopoietic system. It could thus be considered as preleukemic “lesion” promoting age-related myeloid disorders and CHIP, respectively.

Aberrantly high Hh signaling has previously been shown to attenuate DDRs [41–45]. One mechanism for this effect is reduced checkpoint signaling via the ATR/CHK1/p53 axis [42, 43, 45], what we here also observe on the level of CHK1 and p53 activation (Fig. 2). Intriguingly, Gutierrez-Martinez et al. [18] recently reported that aged HSCs display lower ATM-dependent apoptosis-priming as compared to young HSCs resulting in increased colony formation capacity in response to different types of DNA damage, including DSBs. It is conceivable that overactivated Hh signaling in aged hematopoietic progenitors also contributes to a dampening of ATM activity and with that reduced apoptosis in response to DSBs.

Aging seems to differently affect the way HSCs and hematopoietic progenitors handle different classes of DNA damage. The ability to repair DSBs was reported to be unaffected [18]. In contrast, we observed a slowed repair kinetic of bulky adducts in aged Lin- cells. The simultaneous leakiness of cell cycle arrest implies that aged Lin- cells keep on cycling in the presence of bulky DNA lesions. Yet, in contrast to DSBs, cells are equipped with systems that allows tolerance of exactly this class of DNA damage [56]. Bulky adducts largely represent obstacles for processive DNA polymerases that cannot be overcome and potentially lead to destabilization of replication forks. Switch to specialized translesion polymerases of the Y-family allows strand extension over the lesion [56]. Such translesion synthesis (TLS) ensures timely and complete duplication of the genome and avoids prolonged polymerase stalling. However, because translesion polymerases are less faithful than processive polymerases [57], TLS increases the risk of generating mutations. This could especially be of concern in cancer treatment. Chemotherapeutic agents such as cisplatin derivates are used to manage solid tumors, yet can be tolerated via TLS [58]. The mutagenic potential of TLS can therefore be connected to the development of chemoresistance and inhibition of TLS reduces the mutagenic potential of cisplatin treatment [59]. Inhibition of TLS has consequently been proposed to sensitize cancer cells to chemostatic substances and simultaneously avoid induction of mutations that possibly induce chemoresistance [60]. The here reported age-related upregulation of Hh signaling could elevate TLS activity and consequently the mutation frequency specifically in HSCs and progenitors replenishing the myeloid branch. Thus, increased Hh activity would give these cells a proliferative advantage at the cost of increased frequency of mutations, both features known as hallmarks of the aging hematopoietic system. Based on our results, we propose that management of solid tumors in an aged organism via drugs inducing bulky adducts may possibly induce mutations in “by-standing” myeloid-biased HSCs and myeloid progenitors increasing the risk for myeloid disorders. Therefore, simultaneous pharmacologic inhibition of the Hh pathway could be considered to minimize such risks. An attractive additional benefit of such Hh inhibition could possibly be attenuated myeloid-skewed differentiation, restored DDRs and reduced evolution of CHIP, collectively ameliorating aging-associated defects of the hematopoietic system.

## Supplementary information

Supplement
