# Supplementary material for: Elevated Hedgehog activity contributes to attenuated DNA damage responses in aged hematopoietic cells
Source: Leukemia. 2019 Nov 14;34(4):1125–34. doi: 10.1038/s41375-019-0641-3 (PMC7214262; doi:10.1038/s41375-019-0641-3)
Supplement: Supplementary file 1 — Supplement [file 41375_2019_641_MOESM1_ESM.docx]

**Scheffold et al., Supplemental Material**

**Supplemental Methods**

*Animals and cell lines*

Hematopoietic cells were always freshly isolated from mice. Mice were of the C57Bl/6J background and of both sexes, maintained in a pathogen-free facility and fed a standard diet. Young mice had an age of 12-14 weeks, while aged mice were at least 22 months of age. All animal testing was done in accordance with the local authorities of Germany (Ulm University organ removal o.202-1; Baden-Württemberg 35/9185.81-3/919; Thüringen 03-006/13). Lenti-X™ 293T cells (Takara, 632180) were maintained at 37˚C and 5% CO_2_, cultured in DMEM (Sigma) supplemented with 10% FBS (Lonza) and 1 % penicillin and streptomycin (Gibco) and regularly tested on for mycoplasma contamination.

*Enrichment and sorting of hematopoietic cells*

Bone marrow of mice was isolated from tibia, femur, humerus, innominate bone, and spine by crushing bones in a mortar followed by selective lysis of erythrocytes. Lineage negative progenitor cells were enriched by magnetic activated cell sorting (MACS, Miltenyi) using biotin-coupled antibodies against Gr-1 (Biolegend; 108404), TER-119 (Biolegend; 116204), B220 (Biolegend; 103222), CD11b (Biolegend; 101204), CD4 (Biolegend; 100508) and CD8 (Biolegend; 100704) and magnetic anti-Biotin beads (Miltenyi). To sort other hematopoietic cells by flowcytometry, whole bone marrow was first subjected to selective lysis of erythrocytes. c-Kit-expressing cells were then enriched via sequential incubation with an anti–c-Kit-APC antibody (Biolegend; 105812) and magnetic anti-APC beads (Miltenyi), followed by capturing in a LS column placed in a magnetic stand (MACS, Miltenyi). Such enriched cells were then subjected to staining with combinations of antibodies to sort specific subsets of hematopoietic cells according to previously published surface markers (1, 2) and as indicated in Supplemental Figure S1. Cells were first incubated with the same biotinylated antibodies recognizing lineage markers as indicated above.

To sort LT-HSCs as well as myeloid-biased and lymphoid-biased HSCs, cells were then incubated with streptavidin-APC-Cy7 (Biolegend; 405208), anti–Sca-1-PE-Cy7 (Biolegend; 108114), anti–c-Kit-APC (Biolegend; 105812), anti–CD34-FITC (eBioscience; 11-0341-85) and anti–CD150-PE (Biolegend; 115904).

To sort CMPs and CLPs cells were incubated with streptavidin-APC-Cy7 (Biolegend; 405208), anti–Sca-1-PE-Cy7 (Biolegend; 108114), anti–c-Kit-APC (Biolegend; 105812), anti–CD135(Flt3)-PE (Biolegend; 135306), anti–FcγR III/II-PE-Cy7 (Biolegend; 101318), anti–CD127(IL-7Ra)-PerCP-Cy5.5 (Biolegend; 135022), and anti–CD34-FITC (eBioscience; 11-0341-85).

To sort LSK progenitors, cells were incubated with streptavidin-APC-Cy7 (Biolegend; 405208), anti–Sca-1-PE-Cy7 (Biolegend; 108114) and anti–c-Kit-APC (Biolegend; 105812). Sorting was carried out in a FACS AriaIII cell sorter and Diva software (both BD Biosciences). See the supplementary material for the gating strategy (Figure S1). Sorted cells were maintained in serum-free StemSpan SFEM medium (Stem Cell Technologies) supplemented with 50 ng/ml mSCF and mTPO (Peprotech), 1 % penicillin and streptomycin (Gibco).

*Introduction of DNA lesions and colony forming assay*

Cells were extracted from a mouse and divided into two aliquots. One aliquot was mock treated, while cells of the other aliquot were subjected to DNA damage induction. Both cell aliquots were then subjected to same downstream application, such as colony formation studies, mRNA or protein extraction, or DNA repair analysis. Importantly, only cells originating from the same animal were compared to each other in experiments.

For UVC irradiation, cells were washed and resuspended in 1x PBS, transferred to a 12-well plate and irradiated (VL-4.C; LTF Labortechnik). Optionally, cells were pretreated with 5 µM vismodegib (Selleckchem), 2.5 µM cyclopamine (Selleckchem, used in Figure 8c and c’), 2.5 µM cyclopamine-KAAD (Merck-Millipore, used in Figure 8d and d’), or vehicle (DMSO) during 24 h to inhibit Hedgehog activity.

To assess the ability to form colonies, bone marrow cells extracted from a given mouse were stained for the appropriate markers and then divided into two wells. One of the wells was subjected to DNA damage induction such as UVC irradiation (see above) or aldehyde treatment (at 37 ˚C during 4h with 5 mM acetaldehyde (Roth)). Thereafter, cells such as HSCs were sorted individually into wells of 96-well plates containing 100 µl of medium (as defined above). Alternatively, a defined number of cells was sorted into a tube containing medium and then plated into methylcellulose (Mouse Methylcellulose Complete Media; R&D Systems; Cat No# HSC007 (Figures 1 and Supplemental Figure 9); Cat No# HSC006 (Figure 4)). This procedure was chosen for lineage negative cells as well as LSK cells that have been transduced with lentiviral particles for instructed manipulation of the Hh pathway. In the latter case only GFP+-cells were sorted to ensure analysis of positively transduced cells. To study the influence of carboplatin treatment on colony formation, individual HSCs were sorted into wells of 96-well plates containing 100 µl of medium supplemented with 1 µg/ml carboplatin (Sigma). After 10 days, the number of colonies containing 20 cells or more was scored. To correct for different plating efficiencies, the treated samples were compared to their respective mock control originating from the same animal. To study checkpoint function or DNA repair efficiency, cells were maintained in medium at 37 ˚C and lysed or fixed 5 h after irradiation or at indicated time points.

*shRNA constructs and lentiviral production*

shRNA constructs targeting genes of interest were picked from the Expression Arrest pSHAG-MAGIC2 retroviral shRNAmir library (Open Biosystems) and shuttled into the vector SF-LV-shRNA-EGFP (3). Additional shRNA constructs were designed using <http://biodev.extra.cea.fr/DSIR/DSIR.html> and <http://katahdin.mssm.edu/siRNA/RNAi.cgi?type=shRNA> and cloned into the same vector. As a control, an shRNA against luciferase was applied. Following constructs in the miR30 context were used: shLuciferase, TGC TGT TGA CAG TGA GCG CCC GCC TGA AGT CTC TGA TTA ATA GTG AAG CCA CAG ATG TAT TAA TCA GAG ACT TCA GGC GGT TGC CTA CTG CCT CGG A; shSmo_1, TGC TGT TGA CAG TGA GCG AGC TCA GTA TGA GAA GAA GAA ATA GTG AAG CCA CAG ATG TAT TTC TTC TTC TCA TAC TGA GCC TGC CTA CTG CCT CGG A; shSmo_2, TGC TGT TGA CAG TGA GCG AGG TTA TTC TCT TCT ATG TCA ATA GTG AAG CCA CAG ATG TAT TGA CAT AGA AGA GAA TAA CCG TGC CTA CTG CCT CGG A; shPtch1_1, TGC TGT TGA CAG TGA GCG CCA GGT TAC ATG GAT CAG ATA ATA GTG AAG CCA CAG ATG TAT TAT CTG ATC CAT GTA ACC TGT TGC CTA CTG CCT CGG A; shPtch1_2, TGC TGT TGA CAG TGA GCG AAC GAG TGA GTC GAG AAT TAA ATA GTG AAG CCA CAG ATG TAT TTA ATT CTC GAC TCA CTC GTC TGC CTA CTG CCT CGG A.

Lentiviral particles were produced in Lenti-X™ 293T cells (Takara, 632180) by co-transfecting lentiviral vectors carrying shRNA constructs, pCMVdeltaR8.91 and pMD.G following the CaCl_2_ method. Lentiviral particles were concentrated by ultracentrifugation (25000 rpm, 4 h, 4 ˚C) and resuspended in 1x PBS. Transduction of hematopoietic cells with lentiviral particles was carried out in the presence of polybrene (Sigma).

*Western blotting*

Cells were lysed in lysis buffer (2 % SDS, 100 mM Tris pH 6.8, proteinase and phosphatase inhibitors (Complete mini and PhoSTOP, both Roche)). Protein amounts were assessed in a BCA assay (Sigma). Proteins were separated in precast Novex 4%-12% Bis-Tris gradient gels (Life Technologies) and blotted onto nitrocellulose membranes (Bio-Rad). Blocking was carried out with 5% milk in TBST. Following antibodies were used: phospho-Chk1 (Ser345; Cell Signaling, 2341), phospho-p53 (Ser15; Cell Signaling, 12571), cleaved Caspase 3 (Cell Signaling, 9661), and beta-Actin (Sigma, A1978). Bound antibodies were detected with IR-Dye coupled secondary antibodies (Li-Cor; IRDye 680RD Donkey anti-rabbit, 926-68073; IRDye 800CW Donkey anti-mouse, 926-32212) in a Li-Cor Odyssey SA infrared Western blot detection system. Quantification was carried out using Li-Cor software. Scans of whole membranes are shown in the supplementary material (Supplemental Figure 3). Note that after transfer of entire gels, membranes were cut into stripes according to marker bands to allow probing for multiple proteins separated on the identical gel. To allow for sequential detection of different antigens, membranes were stripped with Restore plus WB Stripping buffer (Thermo Fisher).

*Cell cycle analysis*

Lineage negative progenitor cells were washed once in ice-cold 1x PBS, resuspended in 1x PBS, fixed by addition of ethanol, and stored at -20 ˚C during 12 h. Buffer was changed to 1x PBS containing 0.5% Tween 20 (Roth) and supplemented with anti-phosphorylated Histone H3 antibody coupled to APC (BD, 558217). After incubation of 2 h, 15 µg/ml RNase A (Qiagen) was added and the cells were incubated another hour at 37 ˚C. DAPI was added (5 µg/ml) and cells were analyzed in an LSRFortessa using FACS Diva software (both BD Biosciences).

*qPCR*

Total RNA was prepared using either the Qiagen RNeasy mini kit (Qiagen), peqGOLD TriFast (Peqlab), or the TaqMan Gene Expression Cells-to-Ct Kit (Ambion). cDNA was produced with the GoScript Reverse Transcription System (Promega) or the TaqMan Gene Expression Cells-to-Ct Kit (Ambion). Expression levels were analyzed in duplicates or triplicates in an ABI Prism 7500 Sequence Detection system (Applied Biosystems) with Absolute QPCR ROX Mix (ABGene; Thermo Fisher) and the Universal Probe Library system (Roche). Following primers (5’-3’) and probes were used for the individual genes: *p21*, tccacagcgatatccagaca and ggacatcaccaggattggac, probe #21; *Mycn*, cctccggagaggataccttg and tctctacggtgaccacatcg, probe #69; *Ptch1*, tgacaaagccgactacatgc and gtactcgatgggctctgctg, probe #64; *Evc*, gcttgtggcttggctgtc and gagcagacgtttcctgtcatc, probe #50; *Evc2*, tggaggcctacacggaag and ccgcacagcagagtttcat, probe #13; *Gli-1*, ctgactgtgcccgagagtg and cgctgctgcaagaggact, probe #84; *Smo*, gcaagctcgtgctctggt and gggcatgtagacagcacaca, probe #3; *Bcl2*, agtacctgaaccggcatctg and ggggccatatagttccacaaa, probe #75; *Hmbs*, tccctgaaggatgtgcctac and aagggttttcccgtttgc, probe #79; *Polr2a*, gattctggaactcaacactctcc and catcaaccaggtggtacagc, probe #55. Expression levels were compared to either *Hmbs* or *Polr2a* (indicated in figure legends).

*Detection of 6-4 photoproducts*

Lineage negative progenitor cells were washed once in 1x PBS and mock treated or irradiated as described above with 20 J/m^2^ UVC. Cells were then incubated at 37 ˚C and aliquots taken directly after irradiation as well as every two hours post-irradiation. Genomic DNA was prepared from all aliquots by phenol/chloroform extraction. After denaturing, equal amounts of DNA were spotted onto Zeta-Probe GT Blotting membrane (Bio-Rad) using a dot blot device (Bio-Rad). An antibody recognizing 6-4 photoproducts (Cosmo Bio, clone 64M-2, NMDND002) was used to probe for remaining DNA lesions. Bound antibody was detected with a secondary IR-Dye-coupled antibody in a Li-Cor Odyssey SA infrared Western blot detection system. Quantification was carried out using Li-Cor software. Scan of whole membrane is shown in the supplementary material (Supplemental Figure 3).

*Microarray*

We assessed gene expression levels in common myeloid progenitors from three young and three aged mice, respectively, by microarray analysis (G3 Mouse GE 8x60K Microarray Kit; Agilent Technologies, Design ID 028005). Total RNA (100ng) was used in labeling reactions (Low Input Quick Amp Labeling Kit; Agilent Technologies) and slides were scanned using a microarray scanner (G2505C, Agilent Technologies). Expression data is deposited in Gene Expression Omnibus (GSE74093). Initial analysis of gene expression was performed with GeneSpring software (Agilent Technologies).

*Enrichment analysis*

Pre-processing of expression data from our microarray analysis (GSE74093) was performed according to Agilent’s standard workflow. Using 5 quality flags (gIsPosAndSignif, gIsFeatNonUnifOL, gIsWellAboveBG, gIsSaturated, and gIsFeatPopnOL) from the Feature Extraction software output, probes were labeled as detected, not detected, or compromised. Gene expression levels were background corrected, and signals for duplicated probes were summarized by geometric mean of non-compromised probes. After log2 transformation, a percentile shift normalization at the 75% level and a baseline shift to the median baseline of all probes was performed. Enrichment analysis on gene expression data was performed to assess Hh pathway activity in young and aged cells. The Hh gene set is the same as listed Supplemental Figure 4. Mean absolute correlation (Pearson) to class labels of the samples for the genes in the pathway was used as test statistic (4). Computer-intensive Monte-Carlo simulation (via gene sampling) was performed to assess the significance of enrichment of the Hh pathway targets (5). All computations were performed using the R statistical software framework ([http://www.R-project.org](https://xmail.fli-leibniz.de/owa/redir.aspx?C=YNg9MYKmB0WiliX1UrGf7SlbYDbdDdII6FhJZ6Dlu8rLWO1DuHrH5uv-xqc-TOdUAHYVMAvJn2k.&URL=http%3a%2f%2fwww.R-project.org)).

*Metadata analysis*

Published data sets describing the transcriptome of young and old murine HSCs isolated according to different marker combinations were reanalyzed in regard to expression levels of Hh pathway components as well as target genes. Reanalyzed data sets were extracted from Gene Expression Omnibus and were GSE48893 (6), GSE27686 (7), GSE22812478 (8), GSE6503 (9), and GSE4332 (10). Data was screened for differential expression of minimally 1.3-fold (up- and down-regulation) in old HSCs as compared to young controls. All analyzed genes are reported to be connected to the Hh pathway.

*Immunofluorescence*

Long-term HSCs (CD34^-^ LSK) of young mice were isolated as described above, taken up in medium and transduced with lentiviral particles conferring GFP expression as well as a control shRNA (shLuciferase, see above) or an shRNA against *Ptch1* (shPtchd1_1, see above). After 24 h, cells were optionally subjected to UVC irradiation. After 5 hours, cells were spotted onto poly-L-lysine coated diagnostic microscope slides (12-well 5.2mm numbered; Thermo Scientific) and fixed with 4% buffered paraformaldehyde. Cells were then permeabilized in 1x PBS containing 0.5 % Triton X-100 (Roth) and blocked with 2% BSA in 1x PBS. Primary antibodies against p21 (Santa Cruz Biotechnology, clone F-5, sc-6246) or EVC2 (Santa Cruz Biotechnology, sc-28393) were applied overnight. Secondary antibodies anti-mouse-IgG1-Alexa 594 (p21) or anti-goat-IgG Alexa 488 (EVC2) (both Life Technologies) were used to probe for the primary antibodies. DAPI was used to counterstain for nuclei. Expression status of p21 was assessed only in GFP+-cells. Cells transduced with lentiviral constructs were visualized with a Zeiss Imager microscope using oil-immersion 40x and 100x objectives and ZEN software (all Zeiss). To detect EVC2, cells were analyzed with a TCS SP5II confocal microscope with oil-immersion 100x objective and LAS AF software (all Leica). EVC2 expression levels were quantified using ImageJ (version 1.410).

*Bisulfite sequencing*

Lin- cells (10^6^ each) of young and old mice were isolated as described above and genomic DNA prepared by phenol/chloroform extraction. Bisulfite conversion of 500 ng DNA was carried out with the EZ DNA Methylation-Gold Kit (Zymo Research) according to the instruction of the supplier. Genomic regions of interest were amplified with following primers (5’-3’): *Evc*, TGA AGA GAT GTT AGG ATT TTA GAG GGG and CCT CCC CAA ACT TTC AAC CTT TCC; *Evc2*, AAG AGT AGT GGG GGT GAT TTG GAG TGA GG and AAA CTC TTT CAA AAA ATA AAA ACA AAC ACA AAC. PCR fragments were gel-purified with the QIAquick gel extraction kit (Qiagen) and cloned into the pCR-blunt vector (Invitrogen). The inserted fragment was sequenced from at least 10 colonies using a T7 primer. Statistical analysis was carried out with Prism 4 (Graphpad) and the Chi square test was applied.

*Chromatin immunoprecipitation*

Chromatin immuprecipitations were essentially done as described in (11). After washing with 1x PBS, 10^6^ lineage negative progenitor cells were treated during 10 minutes with 1 % formaldehyde (Thermo Scientific), which was quenched by addition of glycine (final concentration 125 mM). Cells were washed twice with 1x PBS. Fixed cells were lysed (1 % SDS, 10 mM EDTA, 50 mM Tris, pH 8.1, 1x Complete –protease inhibitors (Roche)) and subjected to sonication in a M220 Focused-ultrasonicator (Covaris). Fragmentation to 200-250 bp was verified in a Bioanalyzer (Agilent Technologies). Dynabeads Protein A and Protein G (Life Technologies) were mixed 1:1 and pre-adsorbed with 5 mg/ml BSA in 1xPBS, before addition of 2 µg antibody per 30 µl beads (rabbit IgG (Santa Cruz Biotechnology, sc-2027), rabbit-anti-H3K4me3 (Diagenode, C15410030), or rabbit-anti-H3K27me3 (Millipore, 07-449)). After washing off of excess antibody, chromatin fragments were diluted in Buffer Dil (0.01 % SDS, 1.1 % Triton X-100, 1.2 mM EDTA, 16.7 mM Tris pH 8.1, 167 mM NaCl, 1x Complete –protease inhibitors (Roche)) and incubated with beads overnight. Beads were then washed twice with low-salt wash buffer (0.1 % SDS, 1 % Triton X-100, 2 mM EDTA, 20 mM Tris, pH 8.1, 150 mM NaCl) and twice with LiCl wash buffer (350 mM LiCl, 1 % IGEPAL CA630, 1 % deoxycholic acid, 1 mM EDTA, 10 mM Tris, pH 8.1). To reverse crosslinking and elute chromatin, beads were incubated in elution buffer (1 % SDS, 100 mM NaHCO_3_, 250 mM NaCl) at 65 ˚C during 4 h, followed by treatment with Proteinase K (Roche). DNA fragments were retrieved with Agencourt AMPure XP beads (Beckman Coulter). Precipitated promotor sequences were assessed by qPCR in an ABI Prism 7500 Sequence Detection system (Applied Biosystems) with iTAQ Universal SYBR Green Supermix (Bio-Rad) with the use of following primers (5’-3’): *Evc*-Spacer-5’-region, ATGCAAGAGCAAAGGAGCAC and CGCTCAGTGATCACAAAAGAGT; *Evc*-Spacer-3’-region, CAAACAGCGTCTCTGGGAAT and AGGGTGACAAGGGCCTCTA; CTRL-region, GGGGATCCAGTGACCTGAA and AGTTTCGCCTTTGCAGATTC. Enrichment of EVC intergenic spacer regions was assessed by comparison to the CTRL region, which has been chosen according to the Encyclopedia of DNA Elements (ENCODE at UCSC).

*Statistics*

Statistical analysis was performed with Prism 4 and 7 (GraphPad). Applied test, sample sizes, exact p-values as well as measured center and variability are indicated in figure legends or figures directly. Normal distribution of data was tested with Prism 7 applying Shapiro-Wilk normality test. Only Welch’s t test was applied for analysis of two unpaired data samples. Similarity of variance was assessed with Brown-Forsythe test with Prism 7 before applying ANOVA. Data reflecting ratios were log transformed to obtain normal distribution (indicated in figures or figure legends, respectively). For all tests, the α level was set to 0.05. Outliers were identified via ROUT analysis (Q=0.5%) and calculated with Prism 7.

To avoid unjustified weighting of individual mice, exhaustive pairwise comparison was applied in a subset of analysis (11, 12) (Figure 3). In this procedure, all possible pairwise comparisons of individual mice of each age group were calculated and then the ratios compared using a Mann-Whitney test.

**Supplemental Figure 1: Gating strategy for sorting of hematopoietic cells**

Different populations of hematopoietic cells were sorted according to surface markers previously published (1, 2). Representative example of gating strategy to sort following compartments: LSK, CMP, CLP, HSCs, myeloid-biased HSCs (CD150^hi^-HSC) and lymphoid-biased HSCs (CD150^lo^-HSC).

**Supplemental Figure 2: Aging myeloid-biased HSCs loose sensitivity to carboplatin.**

The scatter plots depict the capacity of the indicated types of hematopoietic stem cells from young and old mice to form colonies in response to carboplatin treatment. All cells were freshly isolated, left untreated or treated permanently with 1 µg/ml carboplatin, and growth of colonies was assessed 10 to 14 days later. Shown are (a to b’) absolute numbers of formed colonies as well as the (a” and b”) comparison of colony number between control and treated cells isolated from individual mice. Individual data points represent results obtained with cells isolated from individual mice (biological replicates). Data were log transformed to obtain normal distribution. Black lines: median. Two-tailed unpaired Welch’s t test (a to b’) and two-tailed paired t test (a” and b”). (a, a’, a”) Myeloid (my)-biased HSCs from 5 young and 5 old mice; p=0.0001 (a); p=0.0106 (a’); p=0.0009 (a”). (b, b’, b”) Lymphoid (ly)-biased HSCs from 5 young and 5 old mice; p=0.017 (b), p=0.0092 (b’).


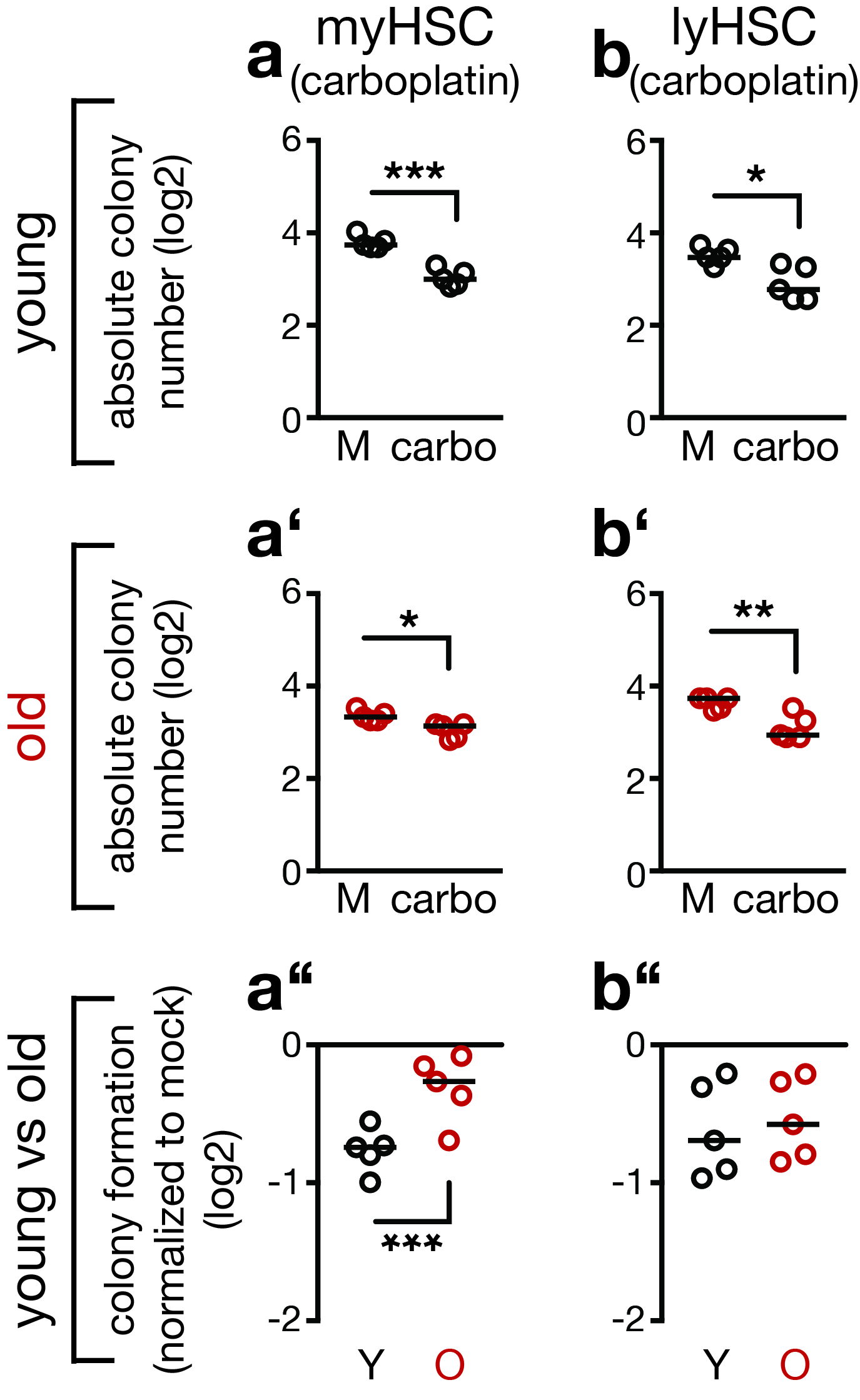


**Supplemental Figure 3: Source file of Western and dot blot.**

(a) Shown is the whole membrane from which relevant bands were quantified and representative areas shown in Figure 2. Cell lysates of individual mice were kept separate to obtain biological replicates. Note that all lysates were loaded onto the same gel. After electrophoresis and transfer of the entire gel, the resulting membrane was cut into stripes according to marker bands to allow probing of multiple antigens of different mass. Arrows indicate bands of interest. Note that the top membrane was first probed for phospho-p53, stripped and then probed for phospho-Chk1.

(b) Shown is the whole membrane of a dot blot from which relevant areas are shown in Figure 2. Arrows indicate the relevant areas (row of dots).


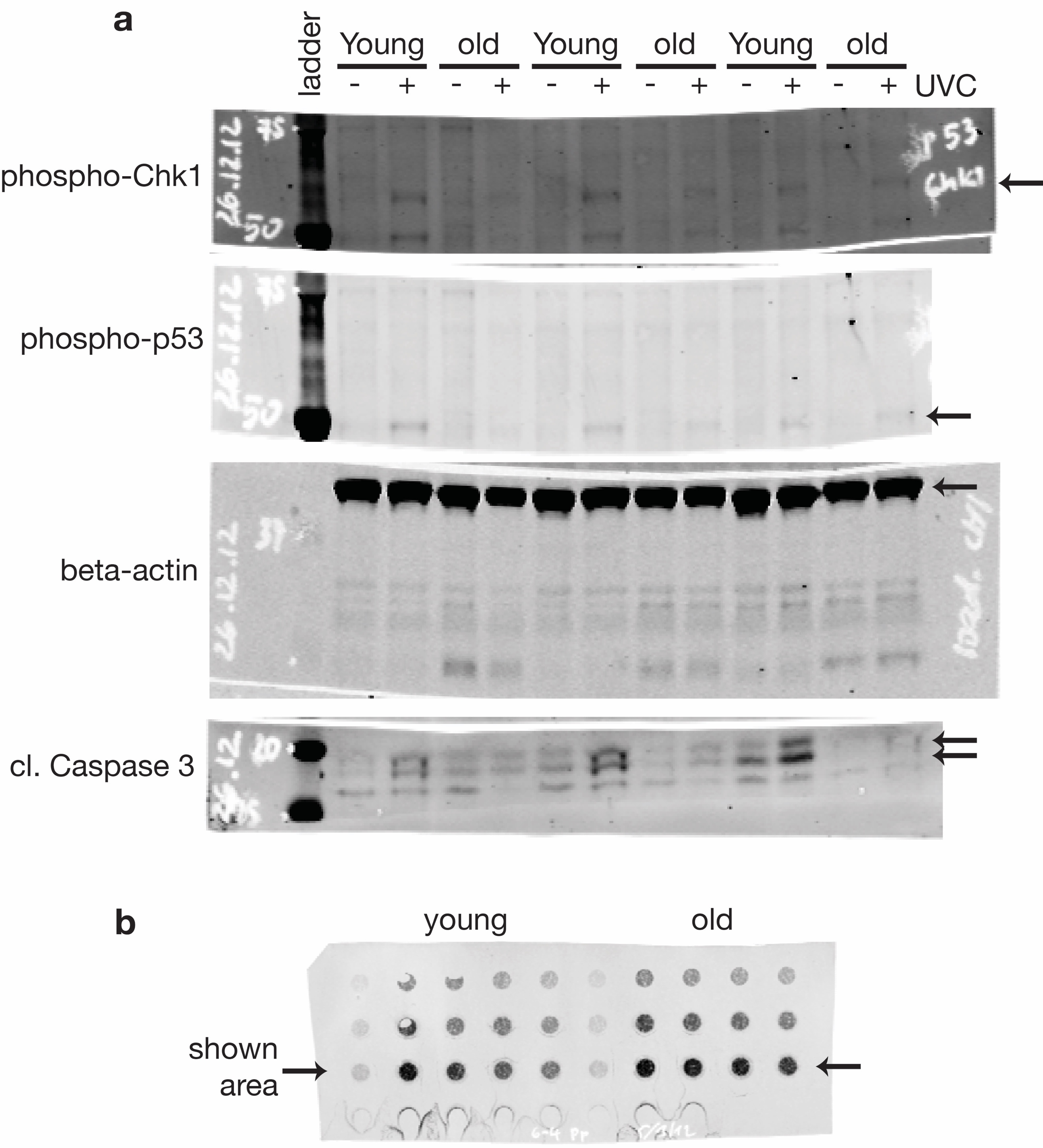


**Supplemental Figure 4: Gating strategy for analysis of cells positive for phosphorylated histone H3.**

Shown are results of mock irradiated lineage negative cells as representative example of gating strategy.


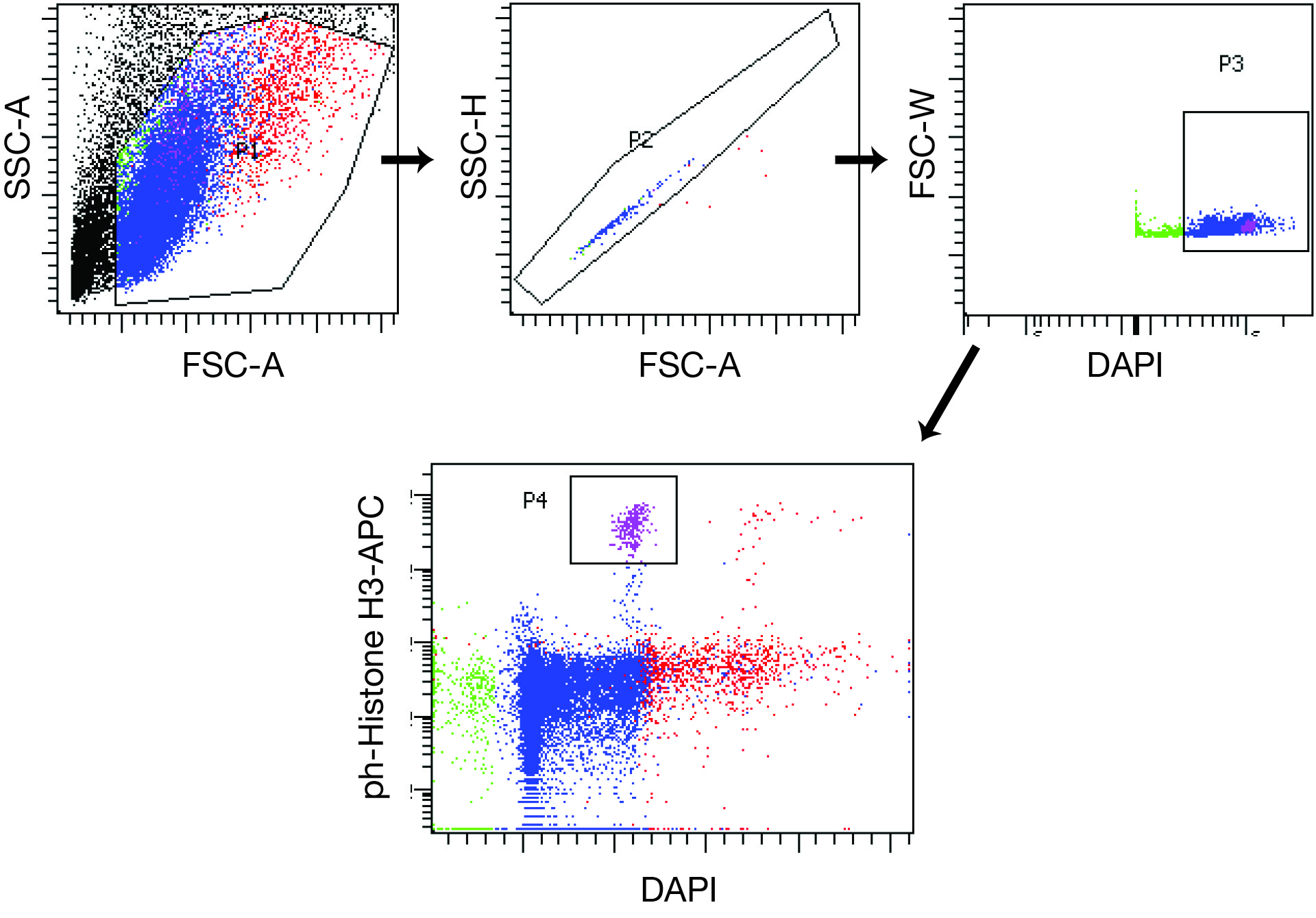


**Supplemental Figure 5: Expression of *Evc* and *Evc2* is increased in hematopoietic progenitors from aged compared to young mice**. (a) Microarray analysis reveals upregulation of *Evc* and *Evc2* in aged common myeloid progenitors (CMPs). Each dot represents a gene. Blue dots indicate *Evc* and *Evc2*, respectively. Additional highly regulated genes are indicated either by their name, or by a letter, which is explained in the panel. Freshly isolated CMPs of three young (3 months) and three old (22 months) mice were analyzed individually. Data are deposited in Gene Expression Omnibus (GSE74093). fs: forward strand; rs: reverse strand.

(b –g) Expression analysis of *Evc* and the *Evc2* in freshly isolated Lin- cells (b, c), CLPs (d, e) and CMPs (f, g) from young and old mice. Expression was normalized to *Hmbs* (Lin- cells) or *Polr2a* (CLP and CMP). Cells of individual mice were analyzed separately and represented as circles. Two-tailed Mann Whitney test (b-e, g) or Welch’s test (f); p values are indicated in figure; n= 4 mice per group in b, c, f and g (young); n= 8 in d and e (both young); n=5 in d and g (old) and n=6 in e (old).

(h, i) *Evc2* protein expression in myeloid- and lymphoid-biased HSCs freshly isolated from young and old mice. Shown are representative examples of myeloid-biased HCSs. For quantification, *Evc2* signals were normalized to the Dapi signals of the same individual cells. N=3 mice per group, myHSC young n=62, old n=61; lyHSC young n=63, old n= 61; Kruskal-Wallis test with Dunn’s correction.


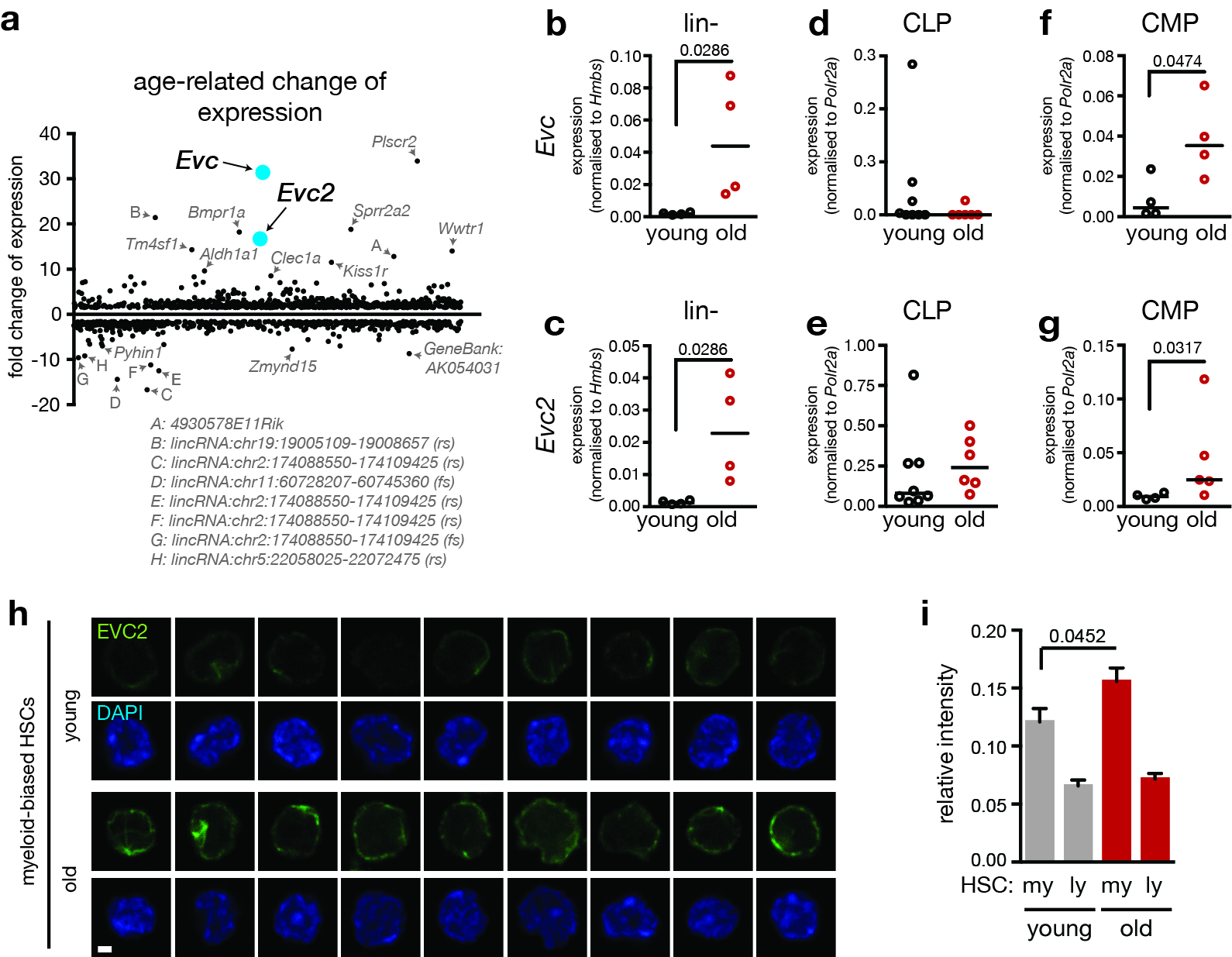


**Supplemental Figure 6: Epigenetic changes induce expression of *Evc* and *Evc2* in aged hematopoietic cells.**

(a) Schematic representation of the genetic locus of the *Evc* and the *Evc2* gene, respectively.

(b) Repressive histone marks (H3K27me3) in the *Evc/Evc2* promoter region are reduced in Lin- cells of aged mice, while active marks are unchanged. Analyzed regions indicated by counter-directional arrows in panel (a). N= 4 mice per group; two-tailed, paired t test on log transformed data; H3K27me3 young vs old, *Evc* p=0.0152, *Evc2* p=0.0092. Mean ±SEM.

(c) Decrease in methylated cytosine in the *Evc*/*Evc2* promoters of aged Lin- cells. Classic bisulfite sequencing was applied to study the methylation status on the level of the genomic DNA in the promoters of the *Evc* and the *Evc2* gene. Schematic representation of classic bisulfite conversion study: filled circles indicate methylated cytosine (meC) while open circles represent unmethylated cytosine. Numbers indicate position relative to the start of transcription.

(d) Quantitative analysis of changes in the methylation status of both promoters as analyzed in (c). n=10, 11; Chi square test; young vs old, *Evc* p<0.0001, *Evc2* p<0.0001.


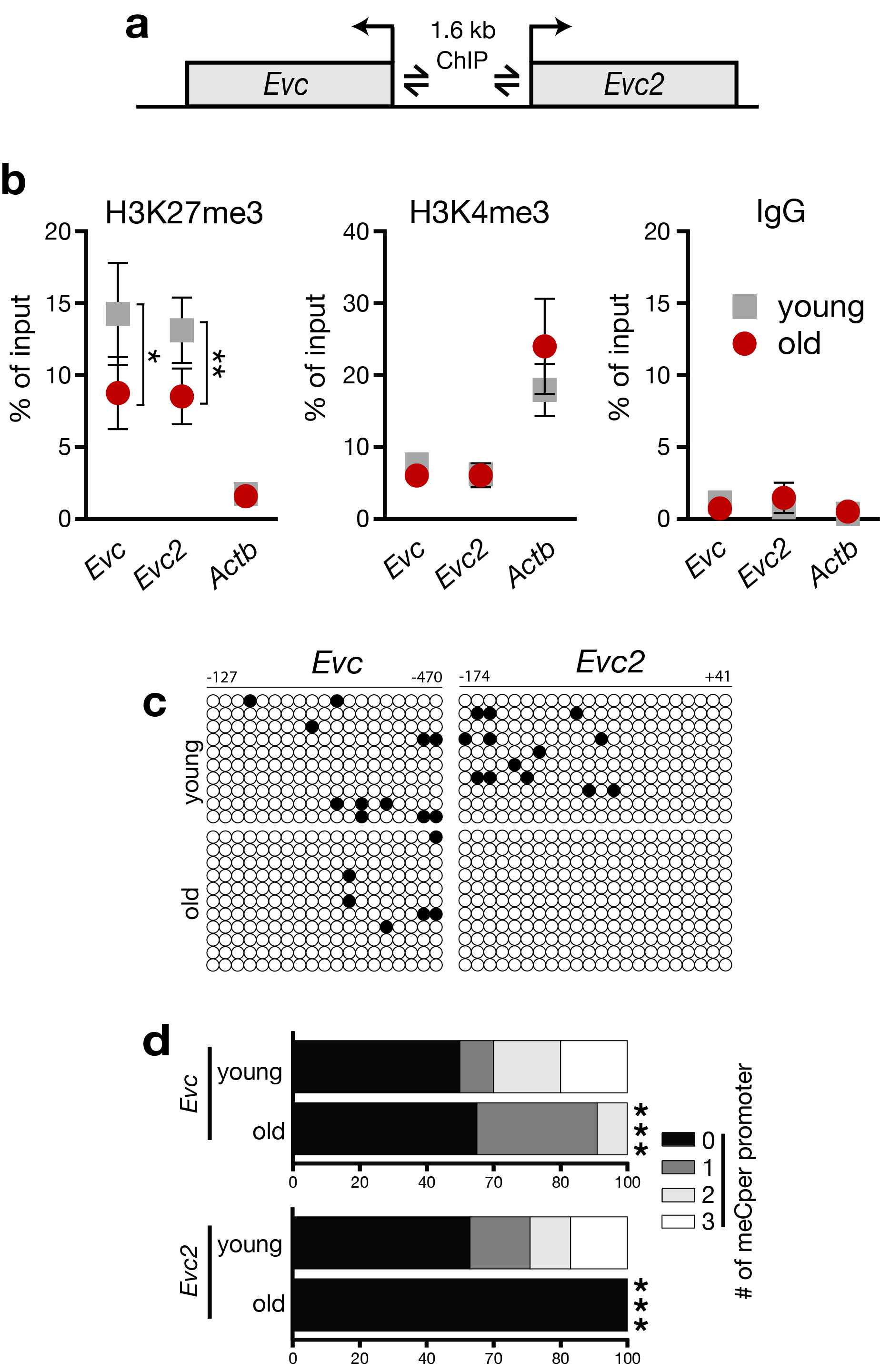


**Supplemental Figure 7: Bioinformatics analysis of Hedgehog pathway activity in aged myeloid hematopoietic stem and progenitor cells.**

(a) Gene set enrichment analysis was performed via a computer intensive re-sampling test (1x10^6^ bootstrap resampling gene sets) to assess whether Hh pathway activity was increased in aged CMPs analyzed by microarray (see Supplemental Figure 5). Lines indicate the 95% quantile from bootstrap resampling (black dotted) and the actual test statistic (t*, red solid) of the representation of genes of the Hh pathway (i.e. p < 0.05). Selected genes are indicated in Supplemental Figure 8 and were chosen due to reported connection to the Hh pathway.

(b) Published transcriptome data of young and old HSCs were re-analyzed in regard to Hh pathway activity with a focus on a set of Hh target genes (see Supplemental Figure 8 for genes). Gene expression differences of >1.3-fold in old versus young HSCs were analyzed. Green dots indicate the number of upregulated genes, while red square indicates the number of downregulated genes. Lines connect numbers obtained from the same data set.


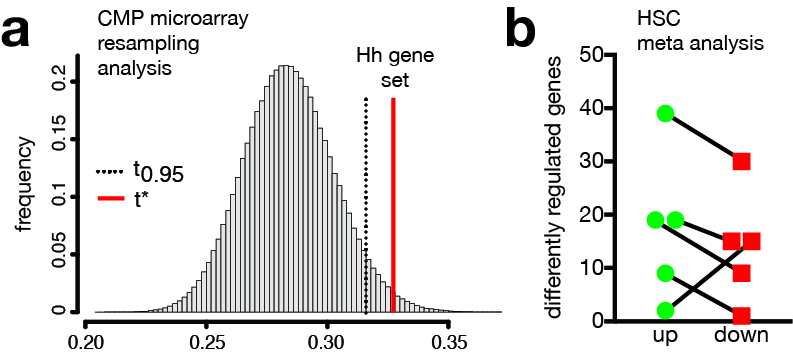


**Supplemental Figure 8: Metadata analysis.**

Published transcriptome data generated from LT-HSCs extracted from young and old mice were reanalyzed concerning expression of Hh components and target genes: GSE48893 (6), GSE27686 (7), GSE22812478 (8), GSE6503 (9), and GSE4332 (10). Analyzed genes are indicated at the bottom, log2FC is given on y-axis. Changes in expression of at least 1.3-fold were considered altered (green=up-regulated, red=down-regulated, grey=unaltered). Note that 4 out 5 studies show predominantly an upregulation of Hh pathway genes.

**Supplemental Figure 9: Hedgehog target genes are upregulated in aged myeloid progenitors.**

To assess Hedgehog pathway activity, expression levels of target genes *Ptch1*, *Mycn*, and *Bcl2* were analyzed in Lin- cells (a), CMPs (b) and CLPs (c).

(a) Expression was normalized to *Hmbs*. *Ptch1* n=8 mice each; p=0.0146; *Mycn* n=4 mice each; p=0.1378; *Bcl2* n=3 and 6 mice, respectively; p=0.0155; (b) n= 4 mice per group, normalization versus Polr2a, (b) *Ptch1* p=0.0212; *Mycn* p=0.1874; *Bcl2* p=0.0443. (c) n=4 mice per group; *Ptch1* p=0.5531; *Mycn* p=0.0053; *Bcl2* p=0.8747. Two-tailed Welch’s test. Cells of individual mice were analyzed in technical duplicates or triplicates and averaged. Circles indicate results from individual mice. Median is indicated by black line.

**
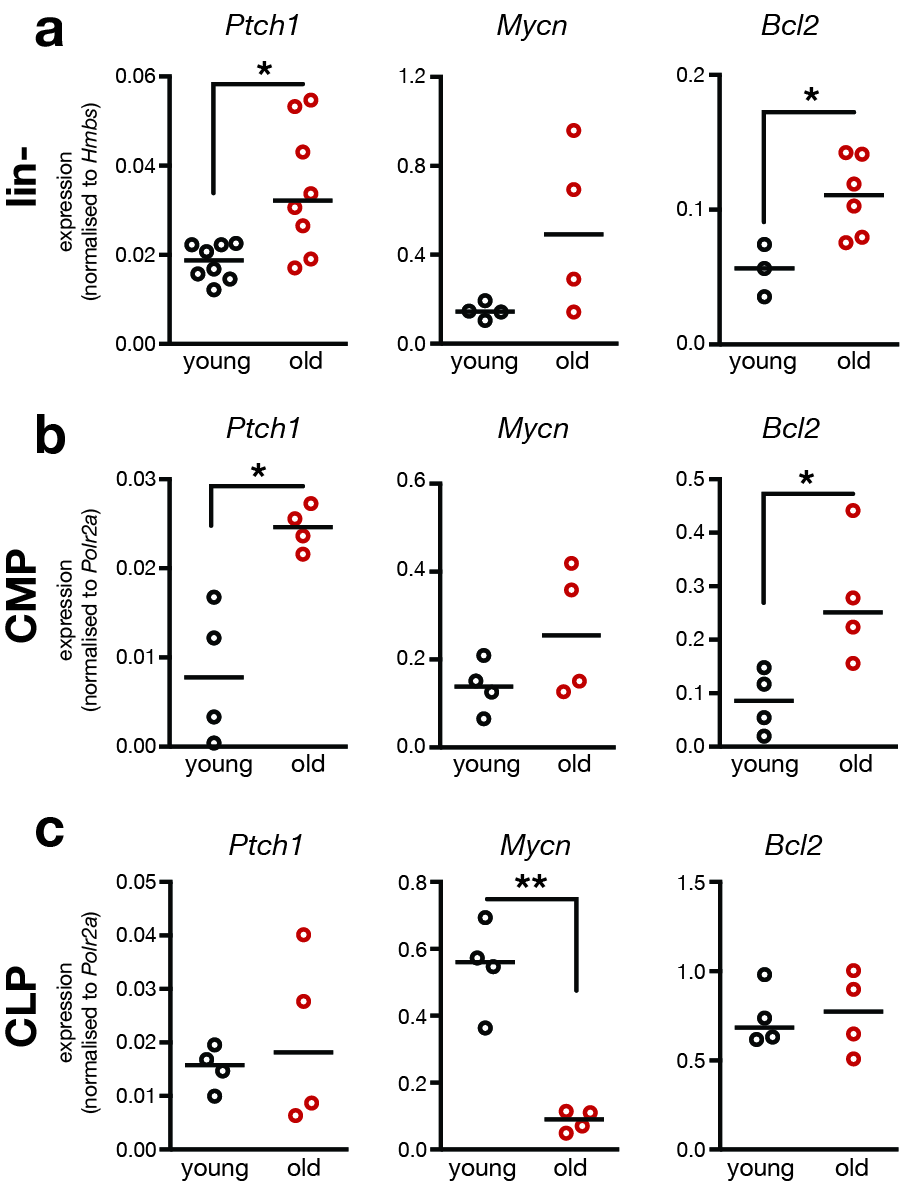
**

**Supplemental Figure 10: Manipulation of Hh signaling via shRNAs alters efficiency of checkpoint functions.**

(a-f) LSK cells from young mice were infected with one of two different shRNAs against *Ptch1* (shPTCH1) or a control shRNA against Luciferase (shLuci), (g-l) LSK cells from old mice were infected with one of two different shRNA against *Smo* (shSMO) or a control shRNA (shLuci). KD efficiency (a, g) and efficiency of Hh manipulation (b, c, h, i) was tested 2 days after virus infection. Changes of expression status (a-c, g-i) was calculated by normalization to average of cells carrying shLuci. The fold induction of *p21* (d, j) was assessed 5 hours after UV irradiation (10 J/m^2^). Also the suppression of colony formation of UV treated (10 J/m^2^; e, k) or acetaldehyde-treated cells (during 4h with 5mM; f, l) relative to mock-control treated cells is shown. Cells treated with DNA damaging agents were compared to control cells isolated from the same mouse. Data points reflect biological replicates and are indicated by circles. Data were log transformed. One-way ANOVA with Holm-Sidak correction (a-d, g, h, j), Kruskal-Wallis with Dunn correction (i), or two-tailed paired t test (e, f, k, l). (a) n=9/6/7, shLuci vs shPTCH1-1 p=0.0032, shLuci vs shPTCH1-2 p=0.0152; (b) n=5/3/8, shLuci vs shPTCH1-1 p=0.0093, shLuci vs shPTCH1-2 p=0.1795; (c) n=9/9/7, shLuci vs shPTCH1-1 p=0.0063, shLuci vs shPTCH1-2 p=0.0088; (d) n=7/3/4, shLuci vs shPTCH1-1 p=0.0002, shLuci vs shPTCH1-2 p=0.0004; (e) n=4, p=0.0075; (f) n=3, p=0.0389; (g) n=6/7/6, shLuci vs shSMO-1 p<0.0001, shLuci vs shSMO-2 p=0.0446; (h) n=6/4/7, shLuci vs shSMO-1 p=0.2187, shLuci vs shSMO-2 p<0.0001; (i) n=6/4/7, shLuci vs shSMO-1 p=0.0349, shLuci vs shSMO-2 p=0.0414; (j) n=8/3/5, shLuci vs shSMO-1 p=0.0201, shLuci vs shSMO-2 p=0.0060; (k) n=3, p=0.0281; (l) n=3, p=0.0325. Cells of individual mice were kept separate and analyzed in technical duplicates or triplicates. Expression was compared to *Hmbs*. Number of biological replicates is indicated above. Median of biological replicates: black line.


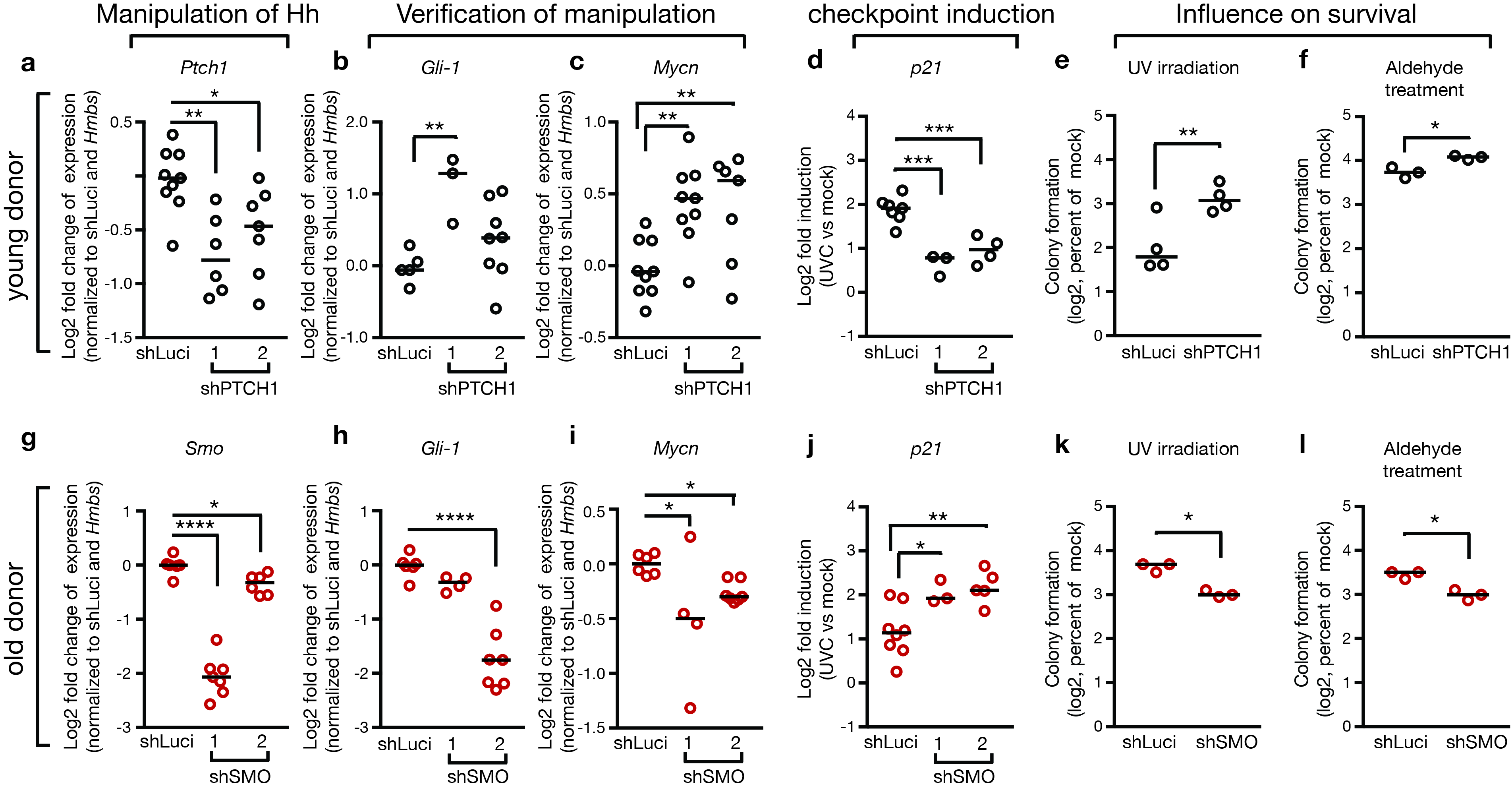


**Supplemental Literature**

1. Beerman I, Bhattacharya D, Zandi S, Sigvardsson M, Weissman IL, Bryder D, et al. Functionally distinct hematopoietic stem cells modulate hematopoietic lineage potential during aging by a mechanism of clonal expansion. Proc Natl Acad Sci U S A. 2010;107(12):5465-70.

2. Morita Y, Ema H, Nakauchi H. Heterogeneity and hierarchy within the most primitive hematopoietic stem cell compartment. J Exp Med. 2010;207(6):1173-82.

3. Wang J, Sun Q, Morita Y, Jiang H, Gross A, Lechel A, et al. A differentiation checkpoint limits hematopoietic stem cell self-renewal in response to DNA damage. Cell. 2012;148(5):1001-14.

4. Ackermann M, Strimmer K. A general modular framework for gene set enrichment analysis. BMC Bioinformatics. 2009;10:47.

5. Katz SF, Lechel A, Obenauf AC, Begus-Nahrmann Y, Kraus JM, Hoffmann EM, et al. Disruption of Trp53 in livers of mice induces formation of carcinomas with bilineal differentiation. Gastroenterology. 2012;142(5):1229-39 e3.

6. Flach J, Bakker ST, Mohrin M, Conroy PC, Pietras EM, Reynaud D, et al. Replication stress is a potent driver of functional decline in ageing haematopoietic stem cells. Nature. 2014;512(7513):198-202.

7. Norddahl GL, Pronk CJ, Wahlestedt M, Sten G, Nygren JM, Ugale A, et al. Accumulating mitochondrial DNA mutations drive premature hematopoietic aging phenotypes distinct from physiological stem cell aging. Cell Stem Cell. 2011;8(5):499-510.

8. Bersenev A, Rozenova K, Balcerek J, Jiang J, Wu C, Tong W. Lnk deficiency partially mitigates hematopoietic stem cell aging. Aging Cell. 2012;11(6):949-59.

9. Chambers SM, Shaw CA, Gatza C, Fisk CJ, Donehower LA, Goodell MA. Aging hematopoietic stem cells decline in function and exhibit epigenetic dysregulation. PLoS Biol. 2007;5(8):e201.

10. Rossi DJ, Bryder D, Zahn JM, Ahlenius H, Sonu R, Wagers AJ, et al. Cell intrinsic alterations underlie hematopoietic stem cell aging. Proc Natl Acad Sci U S A. 2005;102(26):9194-9.

11. Avila AI, Illing A, Becker F, Maerz LD, Morita Y, Philipp M, et al. Xpg limits the expansion of haematopoietic stem and progenitor cells after ionising radiation. Nucleic Acids Res. 2016;44(13):6252-61.

12. Curtiss JH. On the Distribution of the Quotient of Two Chance Variables. The Annals of Mathematical Statistics. 1941;12(4):409-21.
